# Supplementary material for: Are Tourists Facilitators of the Movement of Free-Ranging Dogs?
Source: Animals (Basel). 2022 Dec 16;12(24):3564. doi: 10.3390/ani12243564 (PMC9774271; doi:10.3390/ani12243564)
Supplement: Supplementary file 1 [file animals-12-03564-s001.zip › Figure S1.pdf]

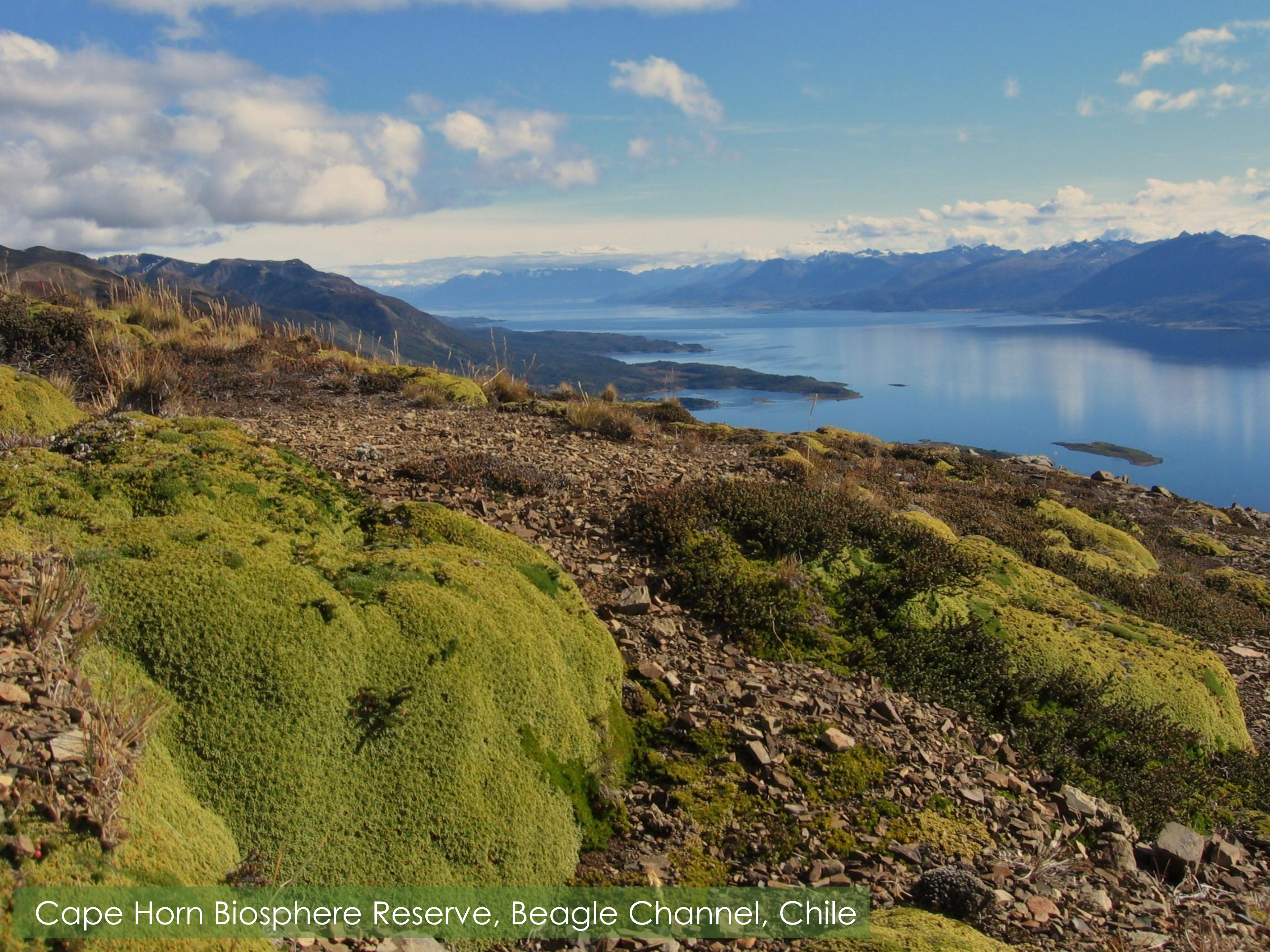

Cape Horn Biosphere Reserve, Beagle Channel, Chile

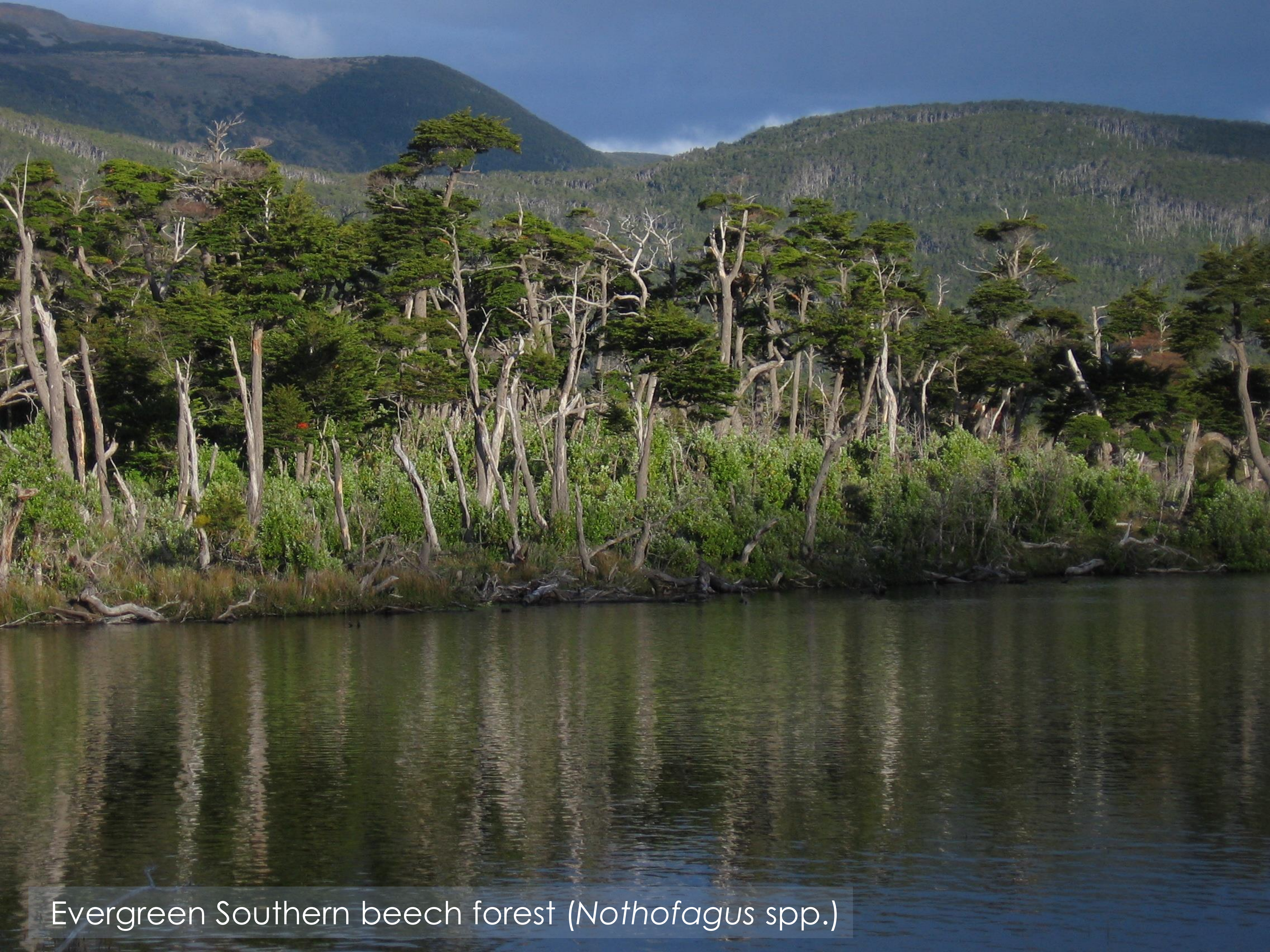

Evergreen Southern beech forest (*Nothofagus* spp.)

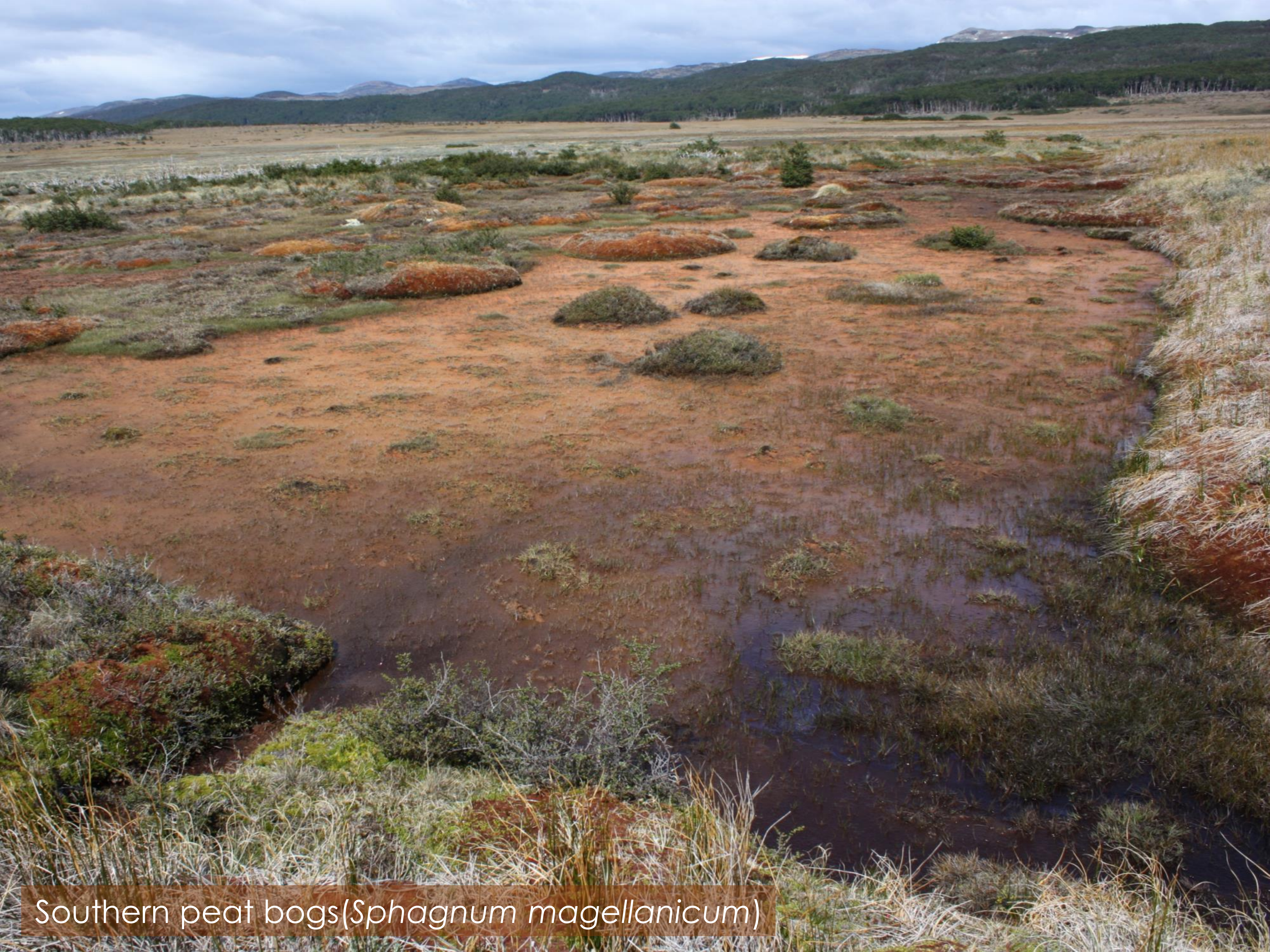
Southern peat bogs(*Sphagnum magellanicum*)

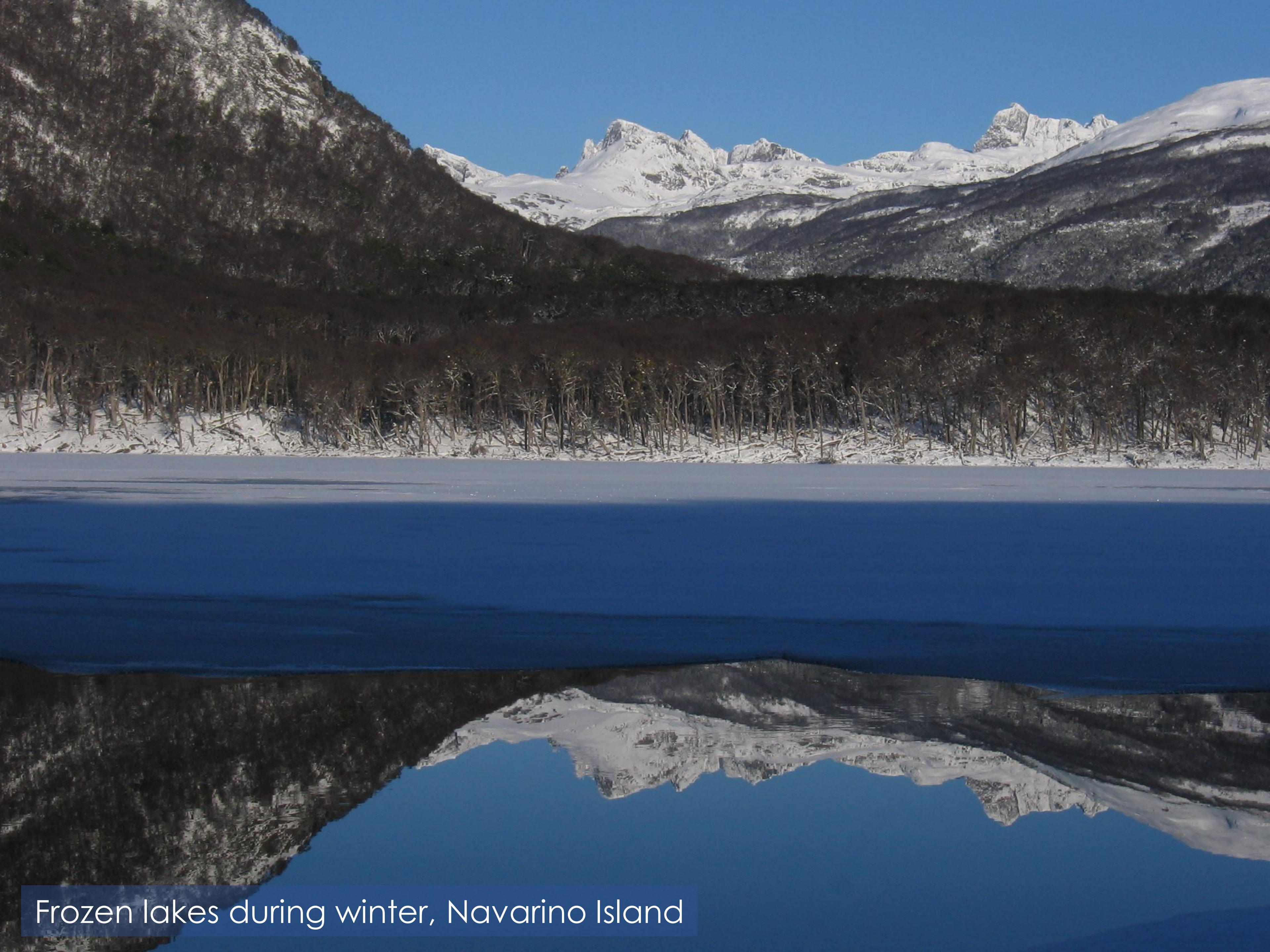

Frozen lakes during winter, Navarino Island

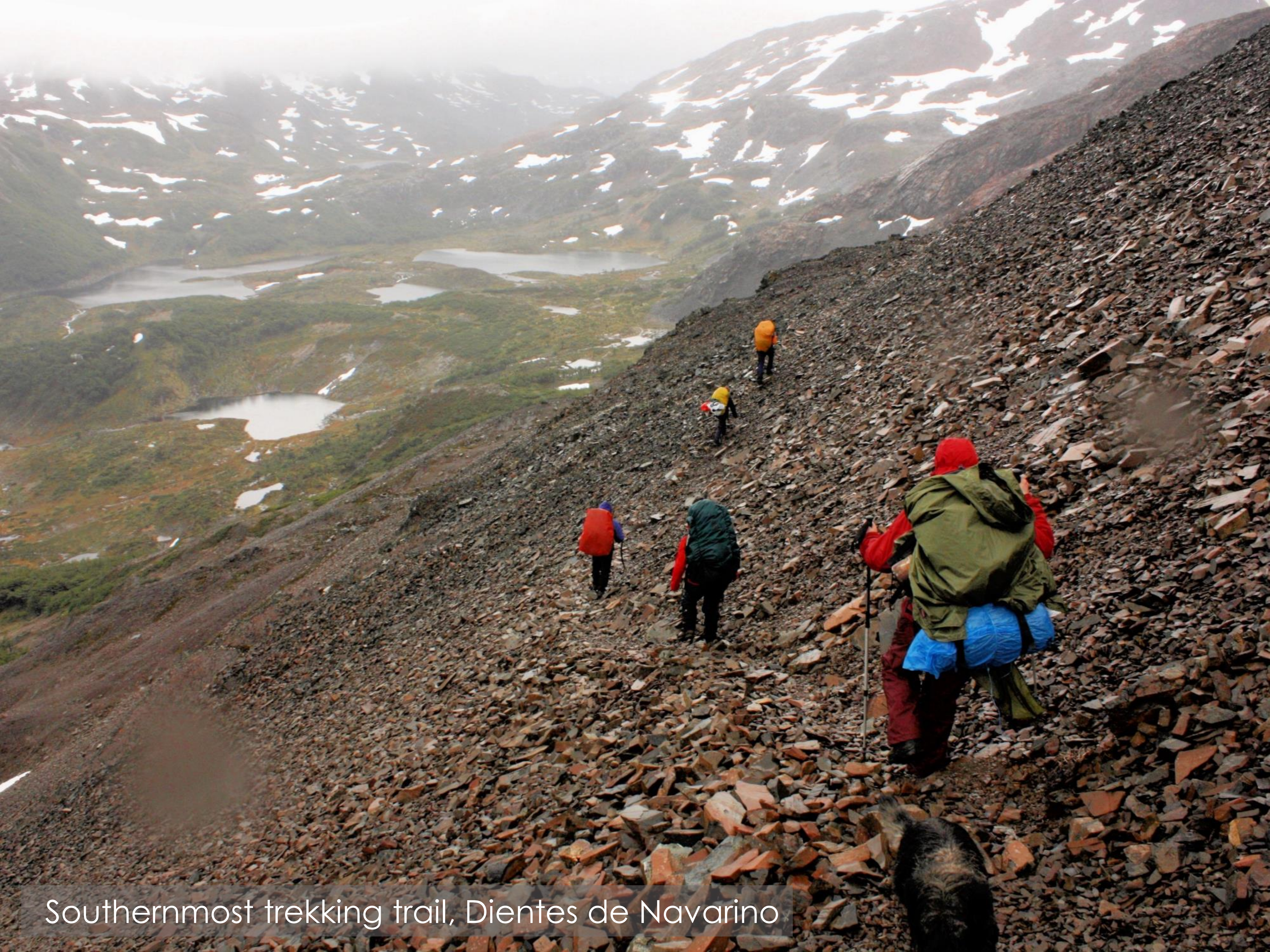

Southernmost trekking trail, Dientes de Navarino
