# Supplementary material for: Are Tourists Facilitators of the Movement of Free-Ranging Dogs?
Source: Animals (Basel). 2022 Dec 16;12(24):3564. doi: 10.3390/ani12243564 (PMC9774271; doi:10.3390/ani12243564)
Supplement: Supplementary file 1 [file animals-12-03564-s001.zip › Figure S2.pdf]

QUESTIONNAIRE FREE-RANGING DOGS
HORN BIOSPHERE RESERVE

A Experience with dogs

1. If you were trekking, did some village dogs follow you? Yes/no
   If yes, how many, for how long, and what do you think why? ____
   ____
2. Did you see dogs chasing another animal during your stay? Yes/no
   If yes, which one(s)? ____
3. Have you fed any dogs during your stay here?
   a) daily
   b) sometimes
   c) never
4. Did you see free-ranging dogs outside the village? Yes/no
   If yes, how many? Where? *Could you please mark the numbers and locations in the map on the next page?*
5. On a scale from 0-10 how afraid are you of encountering free-ranging dogs outside the village (0=no fear, 10=high level of fear)? *Please mark*: 0 1 2 3 4 5 6 7 8 9 10
6. Did you experience problems with free-ranging dogs during your stay here? Yes/no
   If yes, which? ____
   ____
   ____
7. Do you have any other comments?
   ____
   ____

B Personal background

8. Gender: ❑ Female ❑ Male
9. Age: _____
10. Nationality: ______________________
11. Highest completed education level:
    a) none
    b) elementary school
    c) high school
    d) technical/vocational training
    e) University
12. Occupation: ______________________
13. Time spent in Puerto Williams up to date: _________________
14. Reason for your visit of Navarino Island: ____

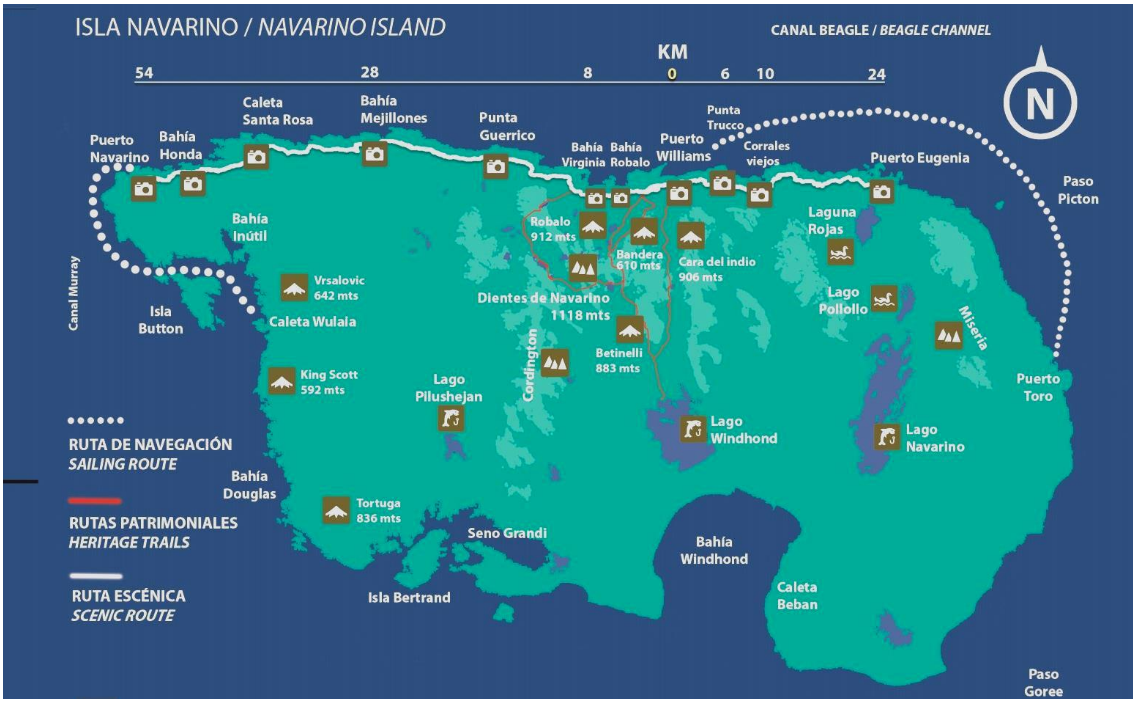
ISLA NAVARINO / NAVARINO ISLAND
CANAL BEAGLE / BEAGLE CHANNEL
KM
54
28
8
0
6
10
24
N
Puerto Navarino
Bahía Honda
Caleta Santa Rosa
Bahía Mejillones
Punta Guerrico
Bahía Virginia
Bahía Robalo
Puerto Williams
Punta Trucco
Corrales viejos
Puerto Eugenia
Paso Picton
Bahía Inútil
Robalo 912 mts
Bandera 610 mts
Cara del indio 906 mts
Laguna Rojas
Lago Pollollo
Vrsalovic 642 mts
Dientes de Navarino 1118 mts
Canal Murray
Isla Button
Caleta Wulaia
Betinelli 883 mts
Misteria
Cordington
King Scott 592 mts
Lago Pilushejan
Lago Windhond
Lago Navarino
Puerto Toro
RUTA DE NAVEGACIÓN
SAILING ROUTE
Bahía Douglas
Tortuga 836 mts
RUTAS PATRIMONIALES
HERITAGE TRAILS
Seno Grandi
Bahía Windhond
Caleta Beban
RUTA ESCÉNICA
SCENIC ROUTE
Isla Bertrand
Paso Goree

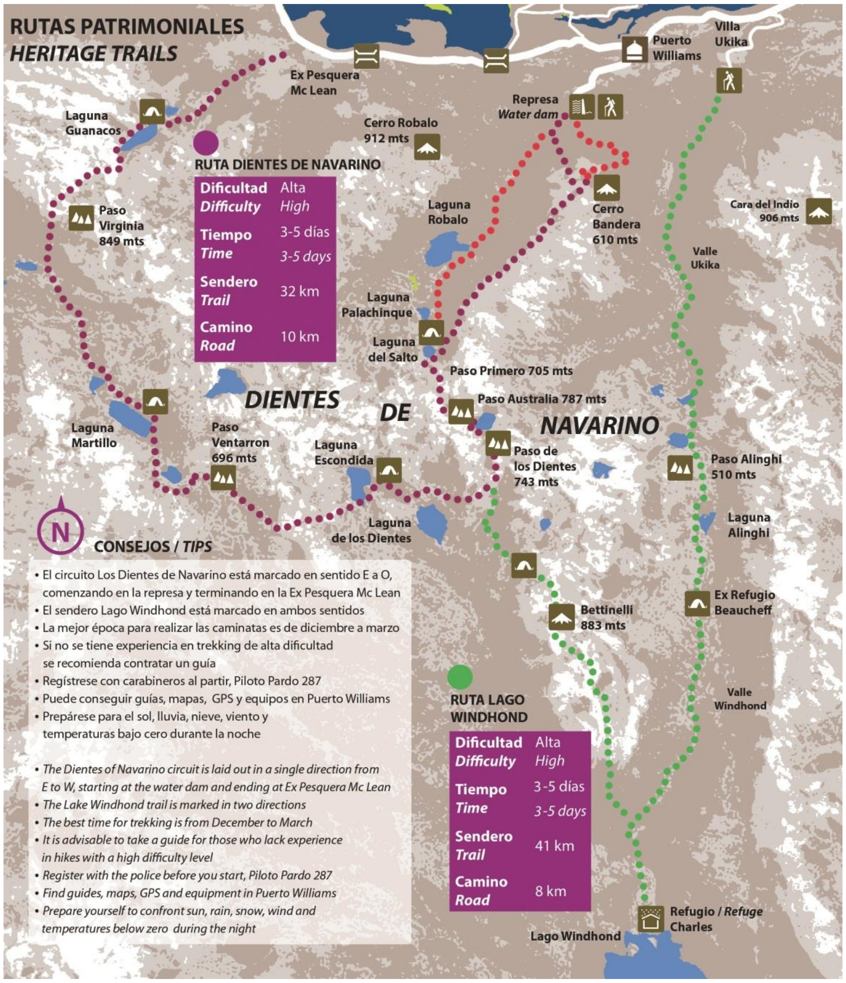
RUTAS PATRIMONIALES
HERITAGE TRAILS
Ex Pesquera Mc Lean
Puerto Williams
Villa Ukika
Laguna Guanacos
Represa Water dam
Cerro Robalo 912 mts
RUTA DIENTES DE NAVARINO
Dificultad Difficulty Alta High
Tiempo Time 3-5 días 3-5 days
Sendero Trail 32 km
Camino Road 10 km
Paso Virginia 849 mts
Laguna Robalo
Cerro Bandera 610 mts
Cara del Indio 906 mts
Valle Ukika
Laguna Palachinque
Laguna del Salto
Paso Primero 705 mts
Paso Australia 787 mts
DIENTES DE NAVARINO
Laguna Martillo
Paso Ventarron 696 mts
Laguna Escondida
Paso de los Dientes 743 mts
Paso Alinghi 510 mts
Laguna de los Dientes
Laguna Alinghi
N
CONSEJOS / TIPS
• El circuito Los Dientes de Navarino está marcado en sentido E a O, comenzando en la represa y terminando en la Ex Pesquera Mc Lean
• El sendero Lago Windhond está marcado en ambos sentidos
• La mejor época para realizar las caminatas es de diciembre a marzo
• Si no se tiene experiencia en trekking de alta dificultad se recomienda contratar un guía
• Regístrese con carabineros al partir, Piloto Pardo 287
• Puede conseguir guías, mapas, GPS y equipos en Puerto Williams
• Prepárese para el sol, lluvia, nieve, viento y temperaturas bajo cero durante la noche
• The Dientes of Navarino circuit is laid out in a single direction from E to W, starting at the water dam and ending at Ex Pesquera Mc Lean
• The Lake Windhond trail is marked in two directions
• The best time for trekking is from December to March
• It is advisable to take a guide for those who lack experience in hikes with a high difficulty level
• Register with the police before you start, Piloto Pardo 287
• Find guides, maps, GPS and equipment in Puerto Williams
• Prepare yourself to confront sun, rain, snow, wind and temperatures below zero during the night
Bettinelli 883 mts
Ex Refugio Beaucheff
Valle Windhond
RUTA LAGO WINDHOND
Dificultad Difficulty Alta High
Tiempo Time 3-5 días 3-5 days
Sendero Trail 41 km
Camino Road 8 km
Refugio / Refuge Charles
Lago Windhond
